# Informal health sector and routine immunization: making the case for harnessing the potentials of patent medicine vendors for the big catch-up to reduce zero-dose children in sub-Saharan Africa

**DOI:** 10.3389/fpubh.2024.1353902

**Published:** 2024-03-07

**Authors:** Abdu A. Adamu, Rabiu I. Jalo, Duduzile Ndwandwe, Charles S. Wiysonge

**Affiliations:** ^1^Cochrane South Africa, South African Medical Research Council, Cape Town, South Africa; ^2^Division of Epidemiology and Biostatistics, Department of Global Health, Faculty of Medicine and Health Sciences, Stellenbosch University, Cape Town, South Africa; ^3^Department of Community Medicine, Bayero University/Aminu Kano Teaching Hospital, Kano, Nigeria; ^4^Vaccine-Preventable Diseases Programme, World Health Organization Regional Office for Africa, Djoué, Brazzaville, Republic of Congo

**Keywords:** zero-dose children, patent medicine vendors, drug shops, hub and spoke, systems thinking, Africa, big catch-up, routine immunization

## Abstract

The COVID-19 pandemic caused a surge in the number of unimmunized and under-immunized children in Africa. The majority of unimmunized (or zero-dose) children live in hard-to-reach rural areas, urban slums, and communities affected by conflict where health facilities are usually unavailable or difficult to access. In these settings, people mostly rely on the informal health sector for essential health services. Therefore, to reduce zero-dose children, it is critical to expand immunization services beyond health facilities to the informal health sector to meet the immunization needs of children in underserved places. In this perspective article, we propose a framework for the expansion of immunization services through the informal health sector as one of the pillars for the big catch-up plan to improve coverage and equity. In African countries like Nigeria, Ethiopia, Tanzania, and the Democratic Republic of Congo, patent medicine vendors serve as an important informal health sector provider group, and thus, they can be engaged to provide immunization services. A hub-and-spoke model can be used to integrate patent medicine vendors into the immunization system. A hub-and-spoke model is a framework for organization design where services that are provided by a central facility (hub) are complimented by secondary sites (spokes) to optimize access to care. Systems thinking approach should guide the design, implementation, and evaluation of this model.

## Background

Immunization is effective in reducing the burden of common vaccine-preventative diseases that affect children, thereby improving their survival and overall development (1). To maximize the benefits of immunization for children, immunization systems worldwide have introduced vaccines such as pneumococcal conjugate vaccine (PCV), rotavirus vaccine, hepatitis B vaccine, and human papillomavirus (HPV) vaccine, among several others, indicating that countries have made progress since the launch of Expanded Program on Immunization (EPI) by the World Health Organization (WHO) 50 years ago (2, 3). It is estimated that immunization averted 39.5 million deaths worldwide (14.4 million in the WHO African Region) between 2011 and 2020, and can potentially prevent another 51 million deaths (23 million in the African region) between 2021 and 2030 (4).

**Figure 1:**
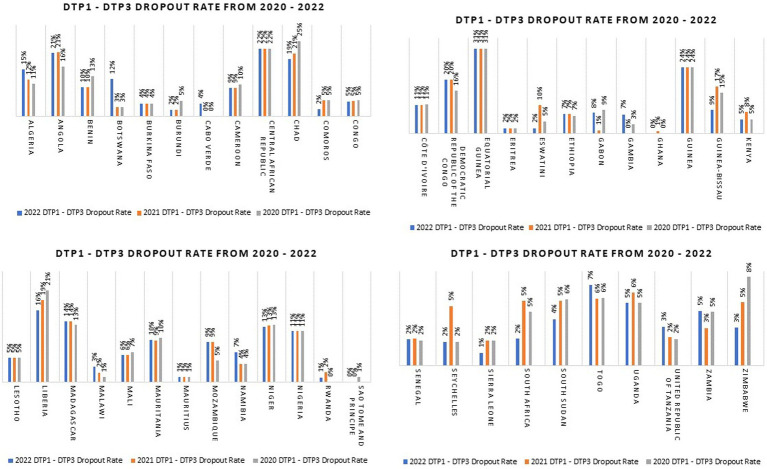
DTP1 – DTP3 dropout rates for countries in the WHO African Region from 2020 to 2022 (Data source: WUENIC 2022) (7).

However, the COVID-19 pandemic severely impacted primary health care systems and disrupted health services, and this affected the performance of immunization programs in Africa and globally in terms of coverage and functionality (5, 6). In the African region, coverage with three doses of the diphtheria-tetanus-pertussis containing vaccines (DTP3) was at 74% in 2016 and 2017 and increased to 77% in 2019 (7). But this marginal progress was lost during COVID-19 as DTP3 coverage declined to 72% in 2021, and still remained at that level in 2022 (7). In addition to decreased coverage levels, millions of children also dropped out from immunization services in Africa during COVID-19. An important measure of dropout, which is used by the immunization program, is the DTP1 – DTP3 dropout rate (8). The DTP1 – DTP3 dropout rate is the proportion of children who took DTP1 but did not complete their vaccination series with DTP3; i.e. under-immunized children (9). This index should not exceed 5% as it reflects the ability of the immunization programs in maintaining access to services (9). An analysis of the 2022 WHO – United Nations Children Fund (UNICEF) Estimates of National Immunization Coverage (WUENIC) data showed that the number of countries with DTP1 - DTP3 dropout rate greater than 5% progressively increased from 22 in 2020 to 23 in 2021 and then to 24 in 2022 (7). In 2022, countries such as Angola, Central African Republic, the Democratic Republic of Congo, Equatorial Guinea, and Guinea have DTP1-DTP3 dropout rate of 20% and above (7). The DTP1 – DTP3 dropout rate for all the countries in the African region from 2020 to 2022 is shown in Figure 1.

The pandemic also affected the coverage level of other antigens (10). The first dose of measles containing vaccine (MCV1) coverage declined from 71% in 2019 to 69% in 2022 and the coverage of the third dose of PCV decreased from 72% in 2019 to 68% in 2022 (7). The disruption of routine immunization services resulted from multiple factors, including government movement restrictions, closure of health facilities, discontinuation of outreach services, and diversion of human resources from essential health care services to COVID-19 outbreak response, among other factors (5, 11, 12).

## Zero-dose children in Africa

Perhaps, the most significant consequence of the COVID-19 pandemic on immunization programs especially in the WHO African Region is the surge in number of unimmunized children, otherwise known as zero-dose children, that it caused (13). Cumulatively, it is estimated that the number of zero-dose children that were added to the African region between 2019 and 2022 is about 28 million (7). These zero-dose children are spread across all countries in the region although the burden varies both between and within countries (14).

One of the immediate health systems implications for having such high numbers of zero-dose children is the occurrence of frequent outbreaks of vaccine-preventable diseases (VPDs) as already observed in some countries in the region (15). In the long term, it can derail progress toward the Immunization Agenda 2030 (IA2030) (16). IA2030 is the global strategic framework for immunization up to 2030 (16). It builds on lessons from the Global Vaccine Action Plan (GVAP) and ambitiously seeks to ensure equitable access to vaccines for all people regardless of their geographical location or age, to maximize the benefits of immunization (16). As part of its strategic priorities, this agenda has set a target to reduce the number of zero-dose children by 50% in 2030, compared to 2019 (13).

## Leveraging the informal sector for immunization

Currently, routine immunization services are mainly provided through fixed sites (which include clinics and hospitals) and outreach stations (17). However, health care facilities are inequitably distributed with prominent disparities between rural and urban communities, and even when they are available, multiple structural factors make them difficult to access (18, 19). The informal health sector caters for the health care needs of people in socioeconomically disadvantaged settings but are rarely engaged to provide immunization services (20). Evidence suggests that zero-dose children are concentrated in such disadvantaged areas, especially rural hard-to-reach communities, urban slums, and communities affected by conflict (14). Therefore, ignoring this sector can delay progress toward reducing zero-dose children as efforts to enhance immunization using the traditional service delivery structures might not improve access where it is most needed.

One important informal health sector provider group that is gaining prominence in public health are patent medicine vendors (21). Patent medicine vendors are individuals who sell medicines in drug shops (21). They are widely distributed in some African countries, mainly in rural areas and urban slums where they serve as the main source of essential healthcare services for the people (22). Some patent medicine vendors have formal health training and even work in hospitals (23). They are often the sole point of contact with healthcare services for people in many underserved communities (21). It is estimated that patent medicine vendors provide 15–83% of child health services in some communities in Africa (24). In some communities in Kenya and Togo, patent medicine vendors treated 69 and 83% of childhood fevers, respectively (25, 26).

There are several reasons for this high patronage of patent medicine vendors. They are often located close to people’s residences, thus eliminating cost of transportation for seeking health care services (24, 25). For example, it is estimated that 87% of rural dwellers in some settings live within 1 km of a patent medicine vendor (25). Furthermore, people perceive services that are provided by patent medicine vendors as quick, and their opening and closing times are flexible, which is convenient (27). Also, they enjoy a high degree of trust from communities and their services are relatively inexpensive or free in some circumstances (28, 29).

Several public health programs are already using patent medicine vendors to improve access to essential services like malaria treatment, family planning including injectable contraceptives, and tuberculosis care (21, 30, 31). Given the spread and penetration of patent medicine vendors in rural communities and urban slums in some African countries, it might be worthwhile for immunization programs to explore opportunities for leveraging them.

## A framework for the big catch-up plan that engages the informal health sector

To address the impact of COVID-19 on immunization system performance, WHO and its partners have launched a recovery plan termed the “Big Catch-Up” (32). The WHO’s Big Catch-Up strategy has three main objectives: “reach children missed during the period 2019–2022 and provide all missing vaccinations; restore vaccination coverage in 2023 to at least 2019 levels; and strengthen immunization systems within primary health care approaches, to improve program resilience and accelerate toward reaching IA2030 and Gavi 5.1 goals and targets” (32).

**Figure 2:**
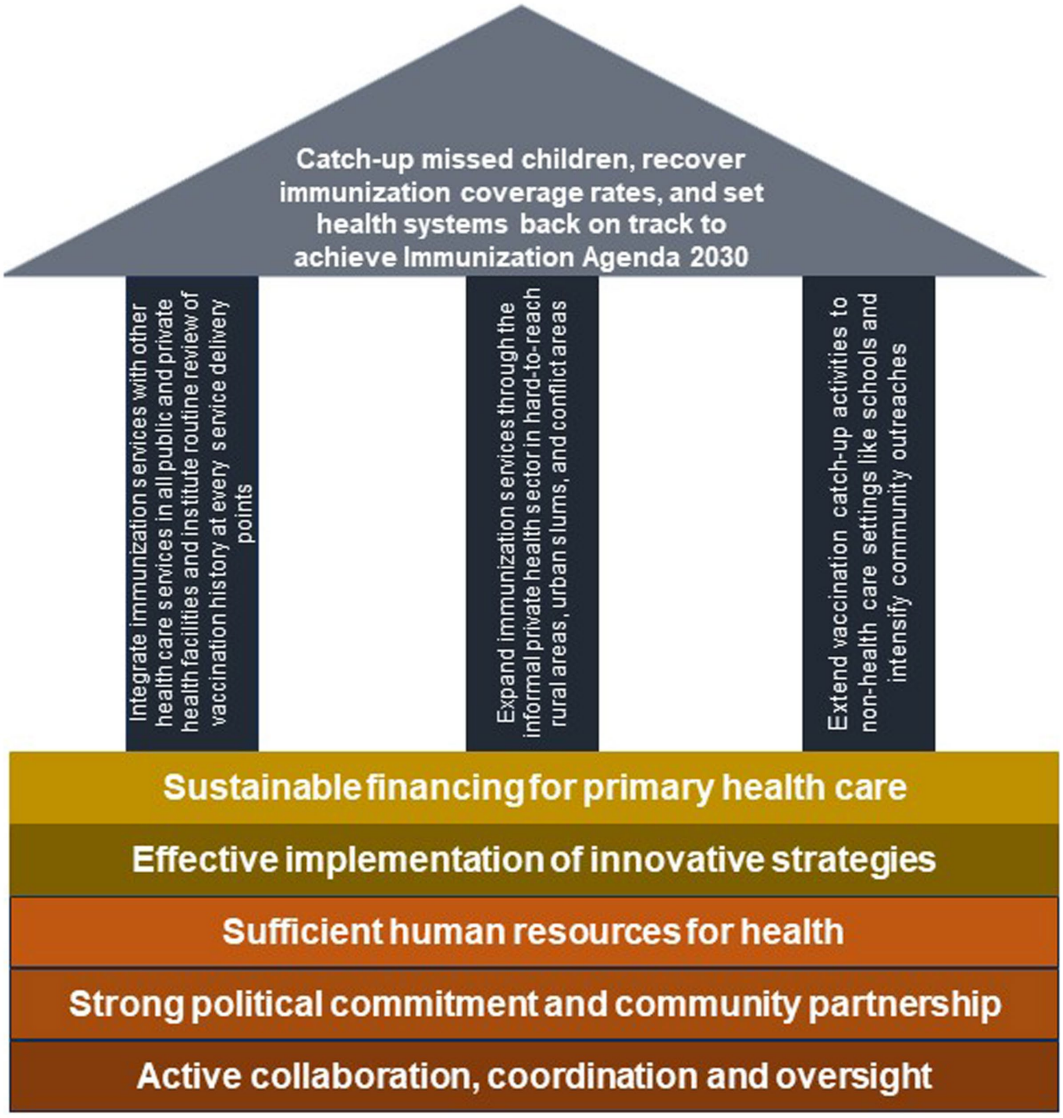
A conceptual framework for the big catch-up.

A conceptual framework that aligns with these objectives and identifies the informal health sector as one of the priorities for the Big Catch-Up is proposed in Figure 2. This framework has three pillars. The first pillar focuses on immunization service integration in public and private facilities, the second pillar is on immunization service expansion through the informal private health sector, and the third pillar is on immunization activities outside the health sector. If immunization service is scaled up to all facilities (both public and private) within a particular area, and health workers at all service delivery points are trained to routinely review vaccination history and immediately vaccinate children who are under-immunized or un-immunized, more children can be reached. Furthermore, if catch up activities are extended to educational settings to target both pre-school children in daycare centers as well as school children, the immunization program is likely to improve in its performance. Additionally, if the informal health sector is integrated into the immunization system to provide immunization services, equity in coverage is likely to improve.

Although countries are expected to urgently initiate actions toward implementing the Big-Catch Up plan, it is important to take into cognizance the underlying complex problems that exist within health systems in the African region. Issues such as inadequate immunization financing, insufficient human resources for health, and weak coordination, among others, are commonly reported challenges (33–35). These constraints can hinder critical activities that are necessary for optimizing the function of immunization programs in the region. Efforts to close the immunization gaps caused by COVID-19 cannot be achieved without substantial financial investment in immunization as well as the other bedrocks highlighted in Figure 2.

## A strategy for integrating patent medicine vendors into the immunization system

A hub-and-spoke model can be used to integrate informal health sector providers like patent medicine vendors into the immunization program to deliver some routine immunization services. The hub-and-spoke model is a framework for organization design where services that are provided by a central facility (hub) are complemented by secondary sites (spokes) to optimize access to care (36). Typically, the range of services that are delivered by the spokes are usually limited in scope, and people who require advanced care are referred to the hub (36, 37). Nevertheless, the spokes are able to leverage the skills and expertise of the hub, and this invariably improves the overall effectiveness of the services provided by the network (36). This model of organizing health care can be used to form a network of patent medicine stores (spokes) around a fixed immunization delivery facility (hub). If designed properly, this model can replicate routine immunization services across multiple hard-to-reach rural communities and urban slums that might not have functional health facilities thereby enhancing access equity (37).

**Figure 3:**
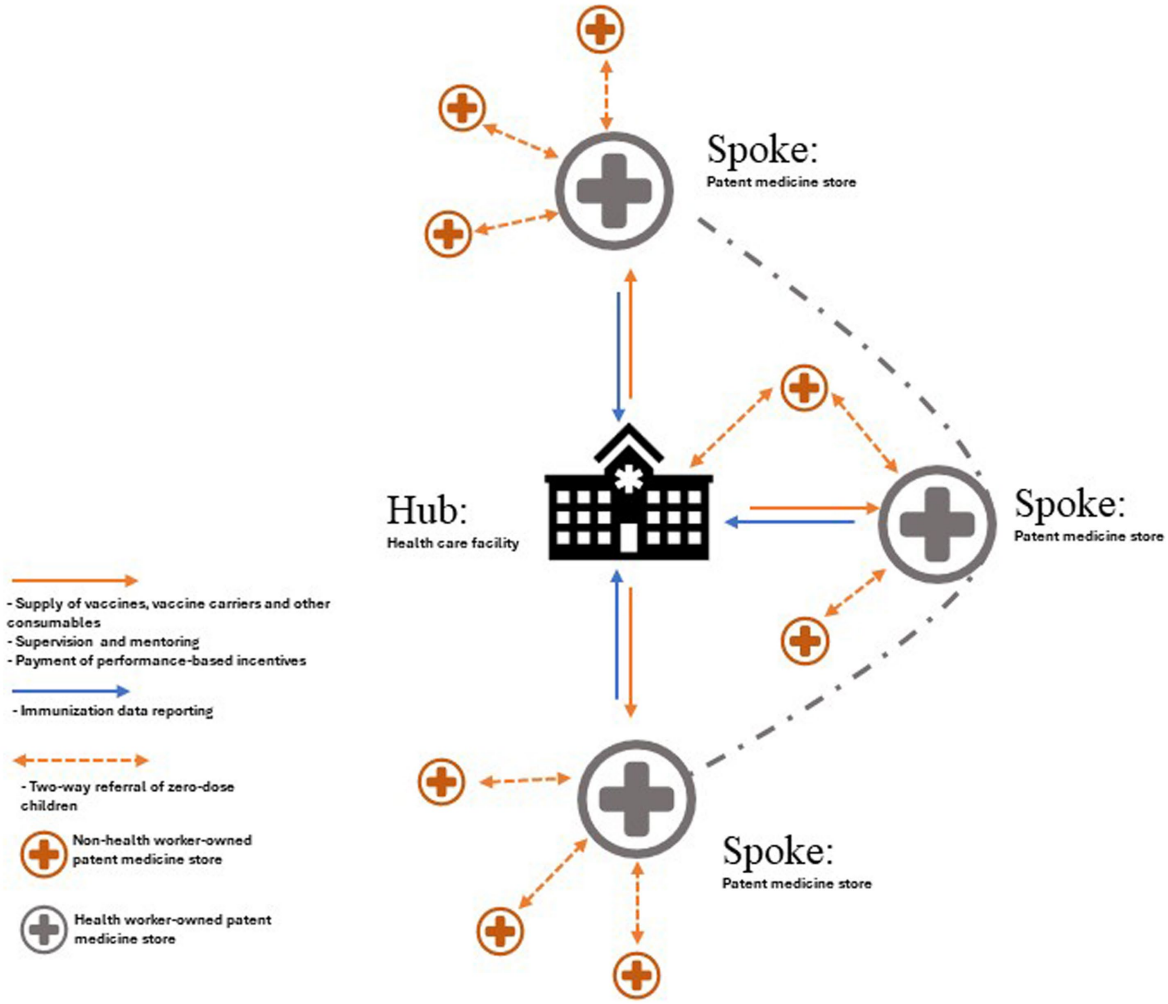
A hub-and-spoke model for integrating patent medicine stores into the existing routine immunization delivery system.

One key drawback that needs to be strongly considered when engaging patent medicine vendors is that managing vaccines is somewhat sophisticated. The medicines that patent medicine vendors typically sell do not have cold chain requirements like vaccines and are easier to store. Nonetheless, this can be addressed through thorough training on immunization including vaccine handling, and the provision of required equipment like vaccine carriers to maintain the cold chain outside the health facility. Another downside to engaging these providers for immunization services is that most vaccines are administered through injections. To overcome this, immunization programs need to be mindful of the roles that are assigned to different types of patent medicine vendors (differentiating those who are trained health workers from those who are not). On a broader level, the immunization programs need to ensure that the regulatory framework that guides the operation of patent medicine vendors is robust, and the quality of public health services that they provide is linked to their re-licensing. Figure 3 is a diagrammatic illustration of a hub-and-spoke network arrangement between a health care facility and patent medicine stores.

In this hub-and-spoke model, the role of a health worker owned patent medicine store (where the patent medicine vendor is a trained health personnel) and a non-health worker owned medicine store (where the patent medicine vendor is not a trained health personnel) is well differentiated as outlined in Table 1. Considering the technical complexity of immunization services, only patent medicine vendors who are qualified health personnel with local authorization to give injections and can recognize adverse events following immunization (AEFI) should be allowed to administer vaccines to children. Other patent medicine vendors who aren’t health personnel are also important as they can be engaged to conduct routine reviews of the vaccination history of children who seek services in their stores to identify under-immunized and un-immunized children, and escort (or refer) them with their caregivers to the nearest facility where they can access immunization services in the communities. This can be at a health worker owned patent medicine store or a health care facility, depending on which is closer to the child. These patent medicine vendors can sustain follow-up for such children until they are fully immunized.

**Table 1:** Potential role of stakeholders involved in an integrated hub-and-spoke model to expand immunization services through patent medicine vendors.

National immunization program	
Coordination, leadership, resource mobilization, and ensuring accountability among all stakeholders in the hub-and-spoke model Development of guidelines for provision of immunization services through patent medicine vendors Identification, engagement, and assignment of differentiated roles for patent medicine vendors based on their background skills Upskilling patent medicine vendors who are trained health workers to provide quality immunization services Training and coaching of patent medicine vendors who are not trained health workers to conduct screening and identification of un-immunized and under-immunized children, and escorting (or referring) and following them up over time until they are fully immunized according to the national immunization schedule. Provision of vaccine carriers to maintain vaccine cold chain High-frequency monitoring and supervision to patent medicine vendors to ensure adherence to guidelines Provision of incentives to hub-and-spoke sites	
Patent medicine vendors who are trained health workers	Patent medicine vendors who are not trained health workers
Reviewing vaccination histories of children who seek health services from them to identify those that are un-immunized and under-immunized. Administration of recommended vaccine doses to eligible children Conducting follow-up reminders until children are fully immunized Conducting regular, small-scale immunization outreach activities within communities Reporting vaccine consumption data to the hub health facility Reporting and referring adverse events following immunization (AEFI) to the health facility using appropriate forms Reporting vaccine hesitancy to the immunization program	Reviewing vaccination histories of children who seek health services from them to identify those that are un-immunized and under-immunized. Escorting (or referring) un-immunized and underimmunized children to the nearest facility where immunization can be offered. Maintaining a register of escorted (or referred) children and sustaining follow-up with them until they are fully immunized. Reporting vaccine hesitancy to the immunization program
Healthcare facilities (hub facilities)	
Store all vaccines at the recommended temperature Ensure distribution of vaccines to designed cold-chain handler of health-worker owned patent medicine stores in vaccine carriers Conduct quality spot checks to patent medicine stores that administer vaccines to ensure that the cold chain is maintained and guidelines are being followed Payment of incentives to patent medicine vendors for the following tasks: Screening, identification, and referral of un-immunized children Administration of vaccines (to be only done by patent medicine vendors that have background health training) Follow-up visits to link eligible children to immunization services Full immunization of each child	

To ensure optimal functioning and effectiveness of the hub-and-spoke model, the national immunization program has important roles to play. This includes coordination and oversight, and building in a clear accountability mechanism for all the stakeholders. A guideline is needed that clearly outlines the functions of all key actors. The immunization program can also develop standard operating procedures that summarize the expected task for each stakeholder. Importantly, all the patent medicine vendors need to be trained by the national immunization program on the role that they are expected to perform. Since patent medicine stores are for-profit entities, providing financial incentives can serve as an important motivator for quality immunization services. The hub facilities should conduct quality spot checks, which should then be used to determine whether a provider qualifies for incentives.

**Figure 4:**
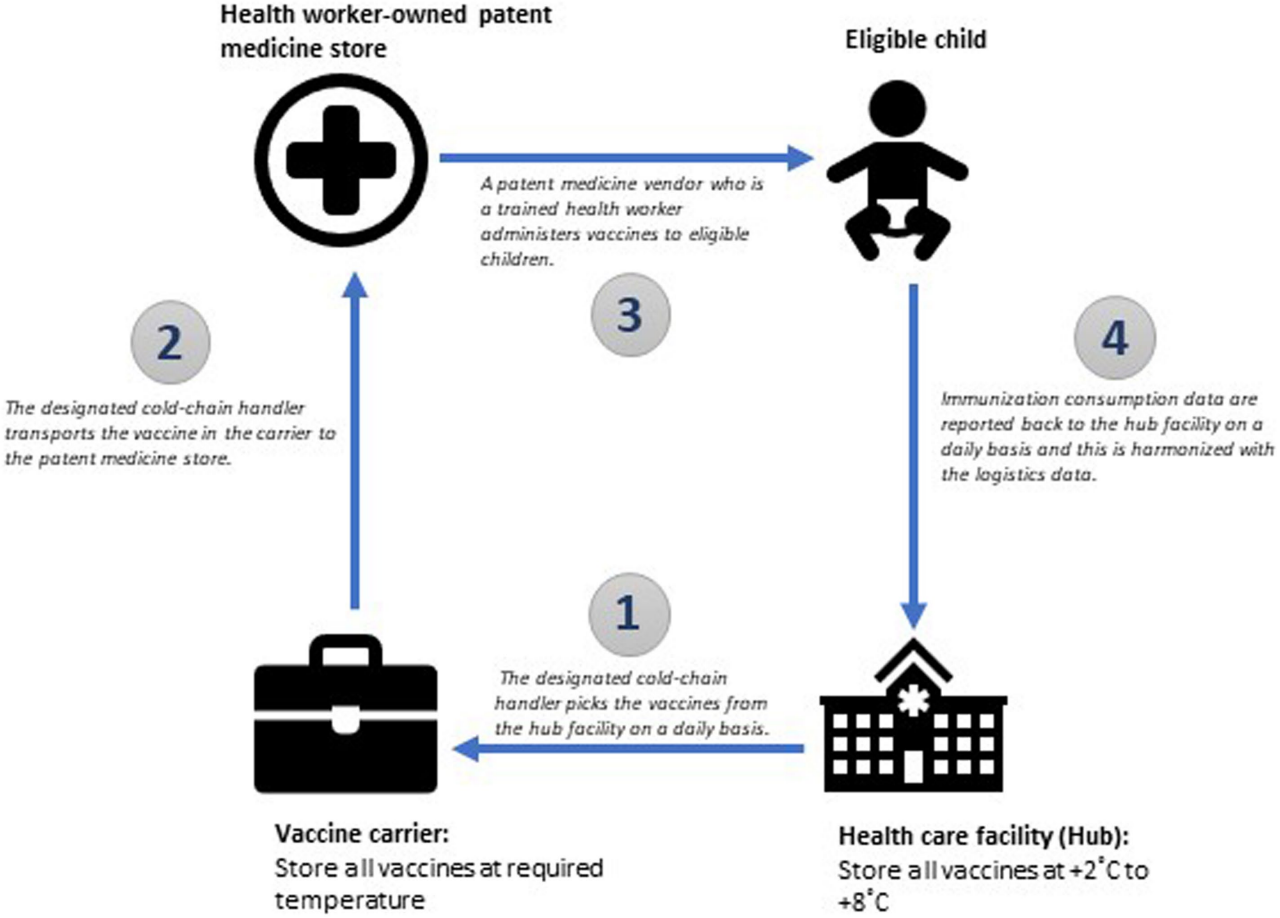
Pathway for transporting vaccines in an integrated hub-and-spoke model.

Vaccines must be stored and transported at the recommended temperature as they move along the pathway from the hub facility to the health worker-owned patent medicine store and finally to the eligible child. The hub facility should have advanced cold chain facilities that are capable of storing vaccines at the recommended temperature for a long period of time. The cold-chain handler at the health facility should be responsible for distributing vaccines to spoke sites through their handler. Once the vaccine is administered, data should be reported back promptly to the hub facility to ensure proper logistic management. The pathway is illustrated in Figure 4.

There are some anticipated benefits of strategically using a hub-and-spoke model in priority zero-dose countries with high volume of patent medicine vendors. They include immunization service expansion in hard-to-reach rural communities and urban slums to meet the needs of children residing in those areas, improvement in immunization knowledge and skills to provide services among patent medicine vendors who are often the first point of contact of health care for many people in underserved areas, and close monitoring and supervision from health workers in the hub facilities. Despite these benefits, some risks should be expected. On the side of the patent medicine vendors, possible risks can include poor quality reporting of immunization consumption data, high workload, poor quality of services, and unauthorized sale of vaccines among others. While on the health facility side, risks can include disruption of vaccine supply including consumables. Measure should be put in place to mitigate these risks in the design phase.

## System-wide implications of integrating patent medicine vendors into the immunization system

Immunization program managers should bear in mind that health care is a complex adaptive system (CAS), thus integrating patent medicine vendors into the immunization sub-system using a hub-and-spoke model will naturally cause the emergence of new connections and the modification of existing ones (38). Typical of CAS is adaptation and self-organization (38). As the system is altered to expand provision of immunization services, these patent medicine vendors will mix into the existing immunization systems (39). Consequently, their behavior will be modulated by already existing agents in the system (such as individuals, health workers, and immunization program managers and policies) through formed dependencies and interconnections (39). Therefore, it is important to apply a systems thinking lens when approaching problems that arise from integrating patent medicine vendors into the immunization sub-system.

Systems thinking tools like causal loop diagrams are useful for exploring the interrelationships and interconnections, including feedback loops that can emerge if patent medicine vendors are integrated into the immunization sub-system (40–42). In a causal loop diagram, arrows are used to show the direction of influence and polarity is denotated using a (+) or (−) sign (41). The feedback loops can either be reinforcing or balancing (41).

## Conclusion

Reducing un-immunized and under-immunized children, restoring immunization coverage to at least pre-pandemic levels, and setting immunization programs back on track from the disruptions caused by COVID-19 require contextualized and tailored approaches. In some communities, engagement of the informal health sector to provide immunization services might be the “magic bullet” that is needed to rapidly improve uptake and utilization of recommended vaccines. Patent medicine vendors are a potential asset within the health systems architecture but remain untapped for routine immunization. Engaging them to provide immunization services can improve access in underserved areas and invariably, improve equity in coverage. For this reason, program managers in countries with high patent medicine vendor activities should strongly consider leveraging them to expand services. However, there is a need to generate empirical evidence on the feasibility and effectiveness of integrating patent medicine vendors into the immunization sub-system using a hub-and-spoke model to reduce un-immunized and under-immunized children. To address this, implementation research in program settings, using type 2 hybrid design, is recommended (43). This type of research is suitable for examining system-wide change in real world implementation context as it allows the assessment of intervention effectiveness as well as implementation outcomes (43). It also enables experiential learning by both researchers and program implementers which allows course correction and can inform scale-up (43, 44).

## Data availability statement

The original contributions presented in the study are included in the article/supplementary material, further inquiries can be directed to the corresponding author.

## Author contributions

AA: Conceptualization, Data curation, Investigation, Project administration, Validation, Visualization, Writing – original draft, Writing – review & editing. RJ: Data curation, Formal analysis, Investigation, Methodology, Validation, Visualization, Writing – review & editing. DN: Data curation, Formal analysis, Investigation, Methodology, Validation, Visualization, Writing – review & editing. CW: Data curation, Formal analysis, Methodology, Supervision, Validation, Visualization, Writing – review & editing.
